# ROS upregulation during the early phase of retroviral infection plays an important role in viral establishment in the host cell

**DOI:** 10.1099/vir.0.055228-0

**Published:** 2013-10

**Authors:** Soo Jin Kim, Paul K. Y. Wong

**Affiliations:** Department of Molecular Carcinogenesis, The University of Texas, MD Anderson Cancer Center, Smithville, TX, USA

## Abstract

Recent studies suggest that low levels of reactive oxygen species (ROS) often modulate normal intracellular signalling pathways, determine cell fates and control cell proliferation. We found that infection of astrocytes with the neuropathogenic retrovirus *ts*1, a mutant of Moloney murine leukemia retrovirus, upregulated ROS at low levels during the early phase of infection. This upregulation of intracellular ROS with downregulation of NADPH levels during the early phase of *ts*1 infection was a separate event from the upregulation of ROS during the late phase while *ts*1-mediated cell death occurred. The treatment of apocynin, a potential inhibitor of NADPH oxidase (NOX), inhibited establishment of the *ts*1 virus in the host cell. These results suggested that ROS generated as a consequence of the activation of NOX may play an important role in the early events of the virus life cycle leading to the establishment of the virus in the host cell. The *in vitro* results were further supported by an *in vivo* experiment which showed that the treatment of apocynin decreased viral titre in the *ts*1-infected mouse brain and increased the lifespan of infected mice. This study provides the first *in vitro* and *in vivo* evidence on a mechanism for how ROS are involved in *ts*1 retrovirus infection and *ts*1-mediated neurodegenerative disease. Our findings focusing on the early phase of the *ts*1 retrovirus life cycle could provide a better understanding of retroviral life cycle, which may offer specific therapeutic targets for suppressing viral replication and alleviating neurodegenerative symptoms in a mouse model.

## Introduction

The retrovirus life cycle is generally divided into two distinct phases: pre- and post-integration phases. The pre-integration phase includes viral entry, reverse transcription and delivery of the pre-integration complex (PIC) carrying proviral DNA to the nucleus for integration into the host genome. The post-integration phase begins with replication and expression of the viral genome, followed by transportation of viral genomic RNA and proteins to the cell membrane for assembly and release of progeny viral particles (Goff, 2004; Nisole & Saïb, 2004). In recent years, intensive research on retrovirus infection, primarily due to the urgency to identify new therapeutic targets against human immunodeficiency virus (HIV) infection, has led to a better understanding of the retrovirus life cycle and host factors involved in retroviral replication (Waheed *et al.*, 2012). However, in contrast to the later events of retrovirus replication, many of the early steps of infection are still poorly understood. Many of the early processes of the life cycle, including reverse transcription and integration, are probably shared by most, if not all, retroviruses. Retrovirus infection often leads to cell activation (Ardavin *et al.*, 1997; Wang *et al.*, 2011) and an increase in intracellular reactive oxygen species (ROS) levels (Pyo *et al.*, 2008; Silic-Benussi *et al.*, 2010). Despite the fact that upregulation of intracellular ROS levels in retrovirus-infected cells and tissues have been well documented, most of these studies assume that retrovirus-mediated ROS upregulation is associated with cell death (reviewed by Wong *et al.*, 2010). In a previous study, we observed that antioxidant treatment ameliorated the effect of the *ts*1-mediated neurodegeneration in the central nervous system (CNS) of infected mice (Jiang *et al.*, 2006). This appeared to be achieved not by removing virus, but by suppressing viral replication in the CNS of infected mice. Moreover, earlier antioxidant treatment after infection was more effective at reducing viral titre in the CNS (Jiang *et al.*, 2006). We suspected that the quiescent CNS environment might be restrictive for productive retroviral replication. To circumvent this restriction, retrovirus might have developed a mechanism to induce intracellular ROS in the host cell as a tool to promote viral propagation.

Cells engage in a variety of ways to modulate levels of ROS to maintain intracellular redox homeostasis. It is important to understand the mechanisms by which cells channel ROS into particular signalling pathways spatially, and/or temporally, to accomplish the desired outcomes. In general, ROS generated by normal cellular processes have been assumed to be unintentional harmful by-products. For example, the mitochondria, where cells produce energy by transferring electrons, have been considered as the major source of intracellular ROS (reviewed by Wong *et al.*, 2010). Cells can also produce ROS intentionally to perform specific functions. A great deal of research has focused on NADPH oxidase (NOX), which transfers singlet electrons to O_2_ to produce O_2_^−^ (Behe & Segal, 2007), because it was the first system identified as an intended ROS generator. The current model of NOX activation was initially based on studies in phagocytic cells, but recent studies have shown that NOX subunits are also present in non-phagocytic cells, including neurons and astrocytes (Behrens *et al.*, 2007; Abramov *et al.*, 2005). ROS produced in non-immune cells may oxidize a more limited spectrum of target molecules, and these specific oxidations can be utilized for particular biological functions rather than producing widespread molecular damage. Upregulation of NOX in activated astrocytes and microglia has been shown to play a pivotal role in HIV infection of the brain and the development of AIDS-associated encephalitis and dementia (Song *et al.*, 2011; Turchan-Cholewo *et al.*, 2009). Whether the induction of intracellular ROS production is involved in the establishment of a retrovirus in host cells remains underexplored. Astrocytes located in the quiescent environment of the CNS are generally not proliferative unless they are activated. Productive infection by retroviruses requires proliferative target cells (Yamashita & Emerman, 2006). We speculated that the *ts*1-mediated upregulation of ROS might activate astrocytes in the CNS, allowing the retrovirus to replicate (Jiang *et al.*, 2006). Despite the established ROS upregulation in *ts*1-infected astrocytes, the source of this ROS and the mechanism of how ROS upregulation contributes to retroviral replication are currently unclear. In this report, results from our *in vitro* experiments suggested that NOX activation is one of the sources of ROS in *ts*1-infected astrocytes and that ROS production played an important role in the early phase of the virus life cycle in the host cell. Consistent with results from our *in vitro* experiments, we observed decreased viral replication, delayed onset of neuropathological symptoms and extended lifespan of *ts*1-infected mice treated with a NOX inhibitor. Together, these results demonstrated that ROS upregulation during the early phase of *ts*1 retroviral infection facilitated its establishment in the host cells *in vivo* and *in vitro*.

## Results

### ROS upregulation during the early phase of infection

**Fig. 1.**
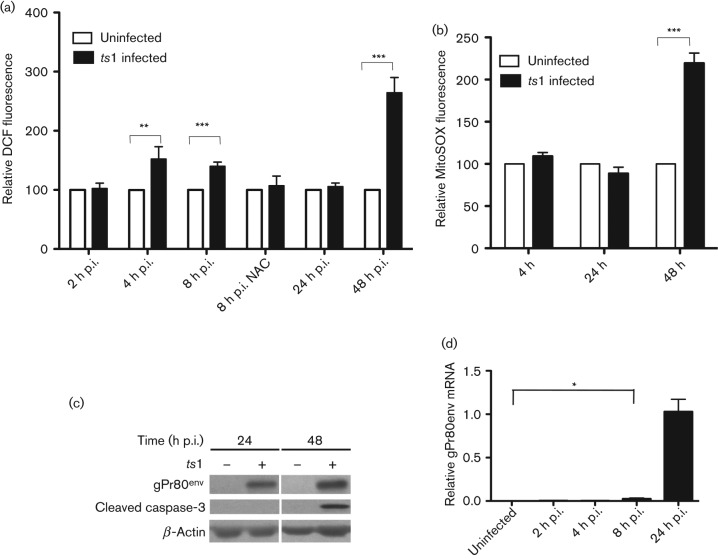
ROS upregulation during the early phase of retroviral infection. (a) Intracellular H_2_O_2_ levels were measured using DCF fluorescence in *ts*1-infected cells. Levels of H_2_O_2_ were compared with uninfected cells. (b) To determine whether upregulated ROS are from mitochondria, mitochondrial O_2_^–^ levels were measured using MitoSOX fluorescence. Upregulation of mitochondrial O_2_^–^ was observed at 48 h p.i., whereas no change was observed at 4 h p.i. (c) *ts*1-mediated apoptosis was verified by detecting caspase-3 activation at 48 h p.i. (d) Viral mRNA was extracted from *ts*1-infected cells at the indicated times and RT-qPCR was performed. Viral gene transcription was detected after 8 h p.i. Statistical significance was determined by Student’s *t*-test: *P<0.05, **P<0.005, ***P<0.0001.

We suspected that ROS upregulation in the early phase of *ts*1 infection might represent a ‘priming’ process to turn on intracellular signal transduction pathways that are necessary for viral establishment in the host genome. To investigate intracellular ROS upregulation in the context of the *ts*1 life cycle, we examined intracellular ROS levels after *ts*1 infection using the fluorescent probes as described in Methods. Infected cells at 4 and 8 h post-infection (p.i.) were shown to have significantly higher H_2_O_2_ levels as compared with uninfected cells, but not at 2 and 24 h p.i. (Fig. 1a). When we treated cells with the antioxidant *N*-acetyl cysteine (NAC), *ts*1-mediated ROS upregulation at 8 h p.i. diminished, suggesting that the dichlorodihydrofluorescein (DCF) fluorescent signals represent intracellular H_2_O_2_ levels. It is noteworthy that we have previously shown that using an m.o.i. of 1–3 for *in vitro* infection of astrocytes, most of the cells are infected (Lin *et al.*, 1997; Shikova *et al.*, 1993). Therefore, with an m.o.i. of 5, what we observed here (from 2 to 48 h p.i.) would be within one life cycle of viral replication, i.e. without secondary or superinfection.

As stated above, upregulation of intracellular ROS levels has been assumed to originate primarily from the mitochondria. To investigate whether this was the case, we incubated *ts*1-infected cells with MitoSOX Red, which selectively targets the mitochondria and is rapidly oxidized by O_2_^−^. As shown in Fig. 1(b), O_2_^−^ upregulation from the mitochondria was detected at 48 h p.i., but not at 4 or 24 h p.i. This suggested that H_2_O_2_ upregulation during the early phase of infection did not originate from mitochondrial O_2_^−^, which occurred at 48 h p.i. during the late phase of infection. At 48 h p.i., activated caspase-3 coincided with O_2_^−^ upregulation, indicating that this O_2_^−^ upregulation was associated with *ts*1-mediated cell death (Fig. 1c). This result is consistent with our previous work showing that *ts*1 infection results in caspase-3 activation and mitochondrial transmembrane potential dissipation (Liu *et al.*, 2004).

To investigate the *ts*1 life cycle, including the process of viral gene transcription after viral DNA integration, we purified viral *env* mRNA from *ts*1-infected immortalized astrocytic C1 cells at 2, 4, 8 and 24 h p.i., and performed reverse transcription-PCR (RT-PCR) using viral *env* gene-specific primers. To quantify RT-PCR products, quantitative PCR (qPCR) was performed. As shown in Fig. 1(d), we detected significant viral *env* mRNA beginning at 8 h p.i., although the levels of viral transcript at this time point were much lower when compared with those at 24 h p.i.

### Apocynin treatment decreased viral DNA after infection

**Fig. 2.**
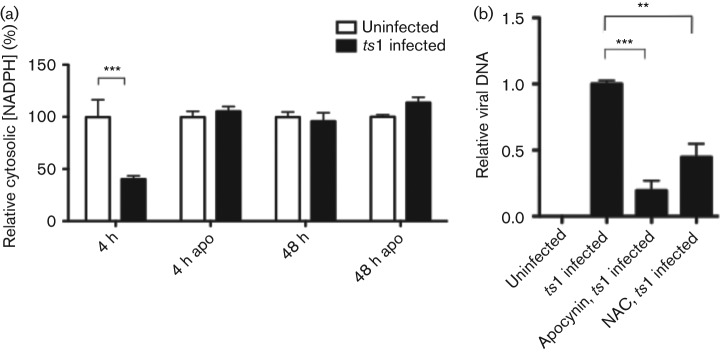
Apocynin (apo) treatment decreased viral gene integration. (a) Intracellular NADPH from whole-cell extracts was measured as described in Methods and compared with uninfected cells. (b) Viral DNA level was quantified using qPCR at 8 h p.i. DNA purified from apocynin- or NAC-treated cells had significantly lower viral *env* gene compared with that from untreated cells. Statistical significance of the results was determined by Student’s *t*-test: *** *P*<0.0001, **P<0.005.

To determine whether the *ts*1-mediated ROS upregulation was mediated by NOX, we attempted to measure NOX activity during the course of viral infection of astrocytes. The activated NOX complex consumes NADPH in the cytoplasm, and transfers electrons to oxygen, thereby generating O_2_^−^. We investigated NOX activity by measuring NADPH. As shown in Fig. 2(a), NADPH levels were decreased at 4 h p.i. However, the decrease of NADPH levels in *ts*1-infected cells was prevented by treatment with the NOX inhibitor apocynin at 4 h p.i. There was no decrease of NADPH levels at 48 h p.i. with or without apocynin. Therefore, the decrease of NADPH level at 4 h p.i. did not appear to reflect a general oxidative status in the infected cells because we did not observe any changes in the levels of NADPH at 48 h p.i. when a high level of ROS was detected (Fig. 1a) during the *ts*1-mediated apoptotic process.

To further investigate whether ROS upregulation during the early phase of infection was involved in early viral establishment in the host genome, we treated *ts*1-infected C1 astrocytes with apocynin or NAC, and then investigated the presence of viral DNA using qPCR analysis. As shown in Fig. 2(b), DNA purified from either apocynin or NAC-treated *ts*1-infected cells contained significantly lower levels of viral *env* gene compared with those from untreated *ts*1-infected cells. It is noticeable that apocynin treatment decreased viral DNA levels more efficiently compared with NAC treatment. Although apocynin could also have antioxidant capacity, our results here suggest the higher efficiency of apocynin, relative to NAC, could be a result of its ability to remove selectively ROS generated via NOX activation rather than a broad-range effect of a general antioxidant, such as NAC.

### Intracellular superoxide dismutase (SOD) activation during the early phase of infection

**Fig. 3.**
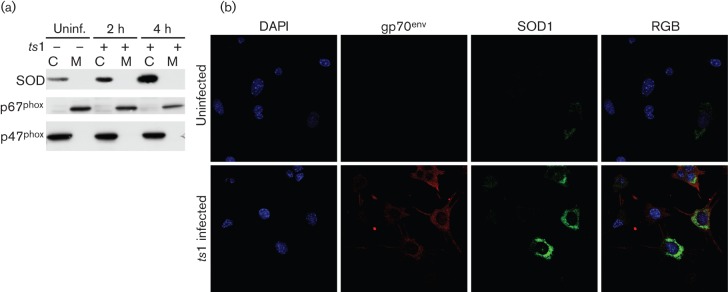
SOD upregulation during the early phase of retroviral infection. (a) The levels of cytoplasmic SOD increased at 4 h p.i. C, Cytoplasmic fraction; M, membranous fraction. (b) The increased cytoplasmic SOD (green) is localized at the perinuclear area of *ts*1-infected cells. To verify infected cells, cells were immunostained with anti-gp70 antibody (red). RGB denotes the merging image of gp70^env^ (red) and SOD1 (green).

It is noteworthy that the levels of ROS and NADPH at 4 h p.i. were detected from the cytoplasm, and that these intracellular ROS levels were likely to come from H_2_O_2_ upregulation. We suspected that at 4 h p.i., intracellular SOD might reduce the upregulated O_2_^−^ immediately by converting O_2_^−^ into H_2_O_2_ after *ts*1 infection. Higher levels of SOD expression occurred in the cytoplasm at 2 and 4 h p.i. (Fig. 3a). To verify the fractionations, we probed for NOX subunits in the cytoplasm (p47^phox^) and membrane (p67^phox^) (Figs 3a and S1, available in JGV Online). Furthermore, upregulated SOD accumulated in the perinuclear area at 4 h p.i. in *ts*1-infected astrocytes whereas SOD in uninfected cells was evenly spread throughout the cytoplasm (Fig. 3b). It appeared that early activation of NOX coincided with the redistribution of SOD in the perinuclear area during the early phase of infection. Along with intracellular ROS upregulation at 4 h p.i., SOD redistribution at the perinuclear area suggested that H_2_O_2_ converted from O_2_^−^ by SOD might allow viral DNA to access the host nucleus or facilitate its integration into the host genome.

### Apocynin rescued cell death *in vitro*

**Fig. 4.**
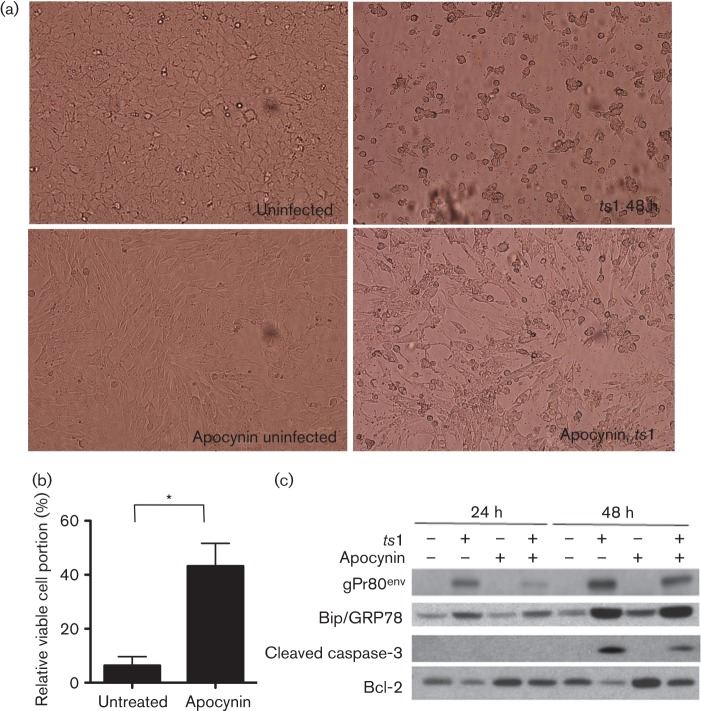
Apocynin rescued *ts*1-induced cell death *in vitro*. C1 astrocytes were infected with *ts*1. (a) One group of cells was cultured in normal medium (top panel) and the other group of cells was cultured 0.5 mM apocynin in the culture medium (second panel). (b) Viable cell numbers were counted from *ts*1-infected astrocyte cultures with or without apocynin treatment, and then compared with cell numbers of uninfected controls. The values of percentage of viable cells are presented. (c) Apocynin treatment increased Bcl-2 and decreased activated caspase-3 levels and gPr80^env^ in *ts*1-infected astrocytes. However, it did not change the expression of binding immunoglobulin protein by apocynin treatment, suggesting that apocynin did not affect ER stress. Statistical significance was determined by Student’s *t*-test: *P<0.05.

*ts*1 infection can cause cell death in astrocytes (Kuang *et al.*, 2010). To investigate whether apocynin treatment could prevent cell death, we counted viable cells of *ts*1-infected astrocytes with or without apocynin treatment. As shown in Fig. 4(a, b), apocynin reduces *ts*1-mediated cell death significantly. Previous work from our laboratory had shown that *ts*1 infection increased caspase-3 activation and decreased Bcl-2 levels in astrocytes (Liu *et al.*, 2004). We questioned whether the apocynin treatment affects *ts*1-mediated cell death after *ts*1 infection. As shown in Fig. 4(c), NOX inhibition increased levels of Bcl-2 and decreased levels of activated caspase-3. We have also previously shown that the neurovirulent effect of *ts*1 is due to the accumulation of misfolded viral *env* protein in the endoplasmic reticulum (ER) resulting in cell death (Kuang *et al.*, 2010). When we treated *ts*1-infected astrocytes with apocynin, we did not see any effect on the level of binding immunoglobulin protein or Bip, which is an indicator of ER stress. These results indicate that *ts*1-mediated NOX activation was not associated with ER stress (Fig. 4c).

### ROS upregulation in *ts*1-infected brain

**Fig. 5.**
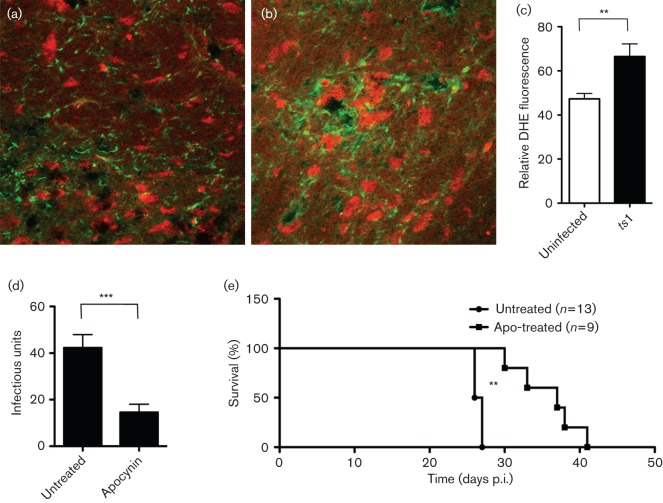
ROS upregulation in *ts*1-infected brain. (a, b) After DHE solution was injected intraperitoneally into both uninfected (left) and *ts*1-infected (right) mice, brainstem tissue sections from the mice were prepared for immunostaining using anti-GFAP antibody. (c) The levels of DHE were quantified using Image J software (each group *n* = 8) and the DHE values of uninfected and *ts*1-infected mouse brains were compared. Statistical significance was determined by Student’s *t*-test. (d) Survival of two groups of mice inoculated with ts1, one with apocynin treatment and the other without treatment. (e) Twenty-two mice were infected with *ts*1. Two days after birth they were divided into two groups. Three days after *ts*1 inoculation, nine mice received intraperitoneal injections of 100 mg apocynin kg^−1^, and the other 13 mice received the same volume of normal saline. Treatment was continued for five continuous days per week, followed by two resting days. After 30 days of infection, brain stems of infected mice were dissected and viral titre assays were performed with the tissues. Lower viral titre was observed in apocynin-treated mice compared with untreated *ts*1-infected mice. Statistical significance was determined by low rank (Mantel–Cox) test. Statistical significance was determined by Student’s *t*-test: ***P<0.0001, **P<0.01.

As previously mentioned, the quiescent environment of the CNS is not favourable for viral replication. To promote viral replication, upregulation of ROS may be required. However, it has not been clear whether ROS increases in the *ts*1-infected mouse CNS. To determine whether NOX is activated in the *ts*1-infected brain, we injected dihydroethidium (DHE) intraperitoneally into both uninfected and infected mice at 25 days after *ts*1 infection. DHE is a cell-permeable probe that undergoes oxidation in the presence of O_2_^−^, leading to the formation of fluorescent ethidium (Behrens *et al.*, 2007). We observed stronger DHE fluorescence in *ts*1-infected brain stems in the area of spongiform lesions compared with normal uninfected brain stems, suggesting that O_2_^−^ production was increased significantly in the *ts*1-infected brainstem (Fig. 5a–c). Although higher DHE fluorescence was detected overall in *ts*1-infected brainstem tissues, it appeared in several distinct spots (Fig. 5b). The subcellular O_2_^−^ detected by DHE appeared to be concentrated in the cell body near the perinuclear area, whereas cytoskeletal glial fibrillary acidic protein (GFAP) was mostly localized at the peripheral regions of the cell (Fig. S2). Viral capsids (p30) were found in both the peripheral regions and the cell body area in astrocytes in the *ts*1-infected mouse CNS (Fig. S2). ROS upregulation was decreased by apocynin treatment (Fig. S3). To validate whether NOX-mediated viral gene integration *in vitro* correlates with the *in vivo* results from the brain, we infected two groups of mice with *ts*1, one with apocynin treatment and the other without treatment. At 30 days p.i., we dissected brain stems from untreated- and apocynin-treated mice and performed viral titre assays. As expected, lower viral titre was observed in apocynin-treated mice compared with untreated *ts*1-infected mice, as shown in Fig. 5(d). We also demonstrated that apocynin treatment extended the lifespan of *ts*1-infected mice, as shown in Fig. 5(e). These results from *in vivo* study suggest that NOX activation is important for early viral establishment in *ts*1-infected cells.

## Discussion

Under physiological conditions, the NOX protein complex is one of the dedicated ROS-generating machineries that regulate many redox-sensitive signalling pathways (Block & Gorin, 2012). Although we observed ROS upregulation in *ts*1-infected astrocytes in our previous study, the mechanism by which *ts*1 increases ROS generation and the exact role that the ROS plays in cellular processes have not been elucidated. In this study, we demonstrated that ROS upregulation during the early phase of *ts*1 infection appeared to be associated with retroviral establishment in the host cell. After penetration of the host plasma membrane through fusion or endocytosis (Miyauchi *et al.*, 2009), the retroviral reverse transcription complex is activated, which is followed by entry of the PIC into the nucleus to assist in viral DNA integration into host genome. The trafficking of retroviral PIC from the plasma membrane to the nucleus is carried out by vesicular structures via the cytoskeletal network rather than by simple diffusion (Naghavi & Goff, 2007). It is noteworthy that at 4 h p.i. by *ts*1, the intracellular ROS level increased as NADPH levels decreased without detection of extracellular ROS upregulation. We suspected that during the early phase of retroviral infection, the membranous compartment in the cytoplasm is probably the place where NOX is activated to generate ROS intracellularly. Given the reactivity of ROS, spatial and temporal regulatory strategies must be present to ensure that a specific type of ROS upregulation occurs only where it is desirable and that the ROS signal is terminated in a timely manner. The advantage of intracellular vesicles is that they can have distinct ROS levels separate from the cytoplasm. High levels of ROS in the vesicles may activate redox-sensitive signalling pathways, while global intracellular ROS concentrations remain at low levels. Oxygen from the extracellular space is secluded into the lumen of early endosomes and converted into O_2_^−^ by the NOX (Oakley *et al.*, 2009). The highly reactive environment of the endosome lumen may play an important role in the activation of the PIC of the invaded retroviruses (Jha *et al.*, 2011). The NOX in the endocytic membrane may upregulate short-lived O_2_^−^ in the lumen, which can be converted into long-lived and diffusible H_2_O_2_ derivatives by SOD, thereby increasing ROS concentration in the microdomains. The upregulated H_2_O_2_ must be sheltered from destruction in selected contexts so that it can perform its intended function. NADPH levels decreased at 4 h p.i., suggesting that NOX activation occurred before viral gene integration. We also observed SOD localization in the perinuclear area within 4 h p.i. We suspect that localization of SOD in the virus-containing endosomes may represent an attempt by the host cell to prevent the pre-integrated viral complex from accessing the nuclear membrane. As shown in Fig. 2(b), inhibition of NOX decreased viral establishment in the host cell. SOD overexpression has been shown to prevent neuronal death in HIV *env*-expressing mouse brain (Louboutin *et al.*, 2007).

Recently, a number of host factors, including generation of ROS, have been implicated in specific steps of virus replication, including virus replication in the CNS. Retroviruses have a long history in neurological diseases (Gonzalesz-Scarano *et al.*, 1995; Power, 2001). Although the *ts*1 mouse model displays more severe neurodegeneration compared with human retroviral disease models, it has several advantages as a retrovirus-mediated neurological disease model. In the *ts*1 model, *in vitro* cellular mechanisms mediated by viral infection have been well-established (reviewed by Wong *et al.*, 2010) and *in vivo* pathological features are highly reproducible and easily identifiable. Our study suggests a link from the ROS upregulation in the cellular levels to the mouse CNS followed by *ts*1 infection. We demonstrated that upregulation of intracellular ROS appears to facilitate retroviral establishment in the host cell. Although our work here focuses primarily on *ts*1 replication in astrocytes, we have previously published work that *ts*1 could infect all cell types in the CNS except neurons (reviewed by Wong & Yuen, 1994). Thus, focusing on astrocytes does not mean that other cells are not involved in *ts*1-mediated neurodegeneration. However, we have previously published a large amount of our work to establish *ts*1’s effect on astrocytes and the role of virus–cell interaction in this cell type in *ts*1-mediated neurodegeneration (reviewed by Wong *et al.*, 2010).

In recent years, certain human neurodegenerative disorders have been linked to human endogenous retroviruses (HERV) (Christensen, 2010). The idea behind this is that HERV genes could re-emerge under specific conditions, such as the result of other viral infections or cell activation. Intracellular ROS upregulation may activate retroviruses. This could give rise to more infectious viruses and spread to other cells in the body, leading to the development of neurodegeneration. It is noteworthy that several other viruses, including human respiratory syncytial virus (Fink *et al.*, 2008) and herpes simplex virus (Hu *et al.*, 2011), have also been shown to induce activation of NO and production of ROS, although the mechanisms underlying the role of NOX and ROS during the life cycle of these viruses are unclear. We believe that understanding the unique CNS environment in the context of virus-mediated upregulation of ROS in the early phase of the viral life cycle and of the host factors involved in virus replication could broaden our knowledge of the involvement of ROS, not only in retrovirus-induced, but also in other virus-induced neurodegenerative diseases. This understanding may also provide insights for antiviral drug targets.

## Methods

### 

#### Virus, cell culture, viral infection of cultured astrocytes and tissue virus titre assay.

*ts*1, a spontaneous temperature-sensitive mutant of Moloney murine leukemia retrovirus, was propagated in a thymus-bone marrow cell line. The titres of viral stock were determined using a modified direct focus assay in the 15F cell line, a murine sarcoma-positive, leukaemia-negative cell line, as described previously (Wong *et al.*, 1985). 15F cells and immortalized murine C1 astrocytes were maintained in Dulbecco’s modified Eagle’s medium (DMEM) supplemented with 10 % FBS, 100 U penicillin ml^−1^, and 100 mg streptomycin ml^−1^ in a 37 °C incubator with 5 % CO_2_ in air. These cells were passed biweekly and used for experiments while in the exponential growth phase. For infection, C1 astrocytes (10^5^ cells) were seeded into 100 mm Petri dishes with one extra Petri dish for counting cell number. On the second day of culture, all cells were treated for 4 h with 3 mg polybrene ml^−1^ and 1 % FBS in DMEM for C1 cells. Cells from one extra Petri dish were then trypsinized and counted. Based on the cell number from counting, *ts*1 virus was diluted in the same medium and added to the cells at an m.o.i. of 5 for C1 cells for 40 min. The cells were washed and incubated with normal culture medium containing 10 % FBS.

The titre (IU ml^−1^) of *ts*1 in brainstems was determined using a modified 15F assay as described above. Brainstems were removed at 30 days p.i., snap frozen in liquid nitrogen and kept frozen until use. Frozen brainstem tissues were weighed and then homogenized (40 strokes; Kontes Glass) in 2 ml ice-cold basal DMEM. Cell debris was removed by filtration through a 0.45 µm syringe filter (Pall Corporation). 15F cells in DMEM medium containing 1 % FBS and 3 µg polybrene ml^−1^ were incubated with diluted tissue lysates for 40 min to allow attachment of virus to the 15F cells. The medium was then replaced with DMEM containing 10 % FBS. The medium was changed on the third day after infection. On the fifth or sixth day after infection, the foci (infectious centres) were counted and the virus titres calculated. Viral titres were calculated as the mean titres from three to six mice.

#### Western blot analysis and subcellular protein fractionation.

C1 astrocytes were washed with PBS and lysed in RIPA buffer as described previously (Jiang *et al.*, 2006). Protein concentrations were determined using a Dc Protein Assay (Bio-Rad Laboratories). The proteins were separated on SDS-PAGE gels, transferred to PVDF membrane and immunoblotted with the indicated primary antibodies. The antibodies used were rabbit anti-cleaved caspase-3 (Cell Signalling), goat anti-gp91^phox^, goat anti-p67^phox^, rabbit anti-p47^phox^ (Santa Cruz Biotechnology), mouse anti-β-actin (Sigma-Aldrich), rabbit anti-SOD (Santa Cruz Biotechnology), goat anti-gp70^env^ (Kuang *et al.*, 2010) and rabbit anti-p30^capsid^ (kindly provided by the Program Resources and Logistics Branch of NCI). After *ts*1 infection, C1 cells were harvested for fractionation and the cell lysates were separated into four subcellular fractions (cytoplasmic, membrane, nuclear soluble and chromatin-bound protein extracts) in accordance with manufacturer’s protocol (Thermo Scientific). In brief, cytoplasmic fractions were collected after plasma membrane permeabilization. Membrane compartments (plasma membrane and organelle membranes) were then dissolved in the membrane fraction. After recovering intact nuclei by centrifugation, the agent yielded soluble nuclear extract, and then chromatin-bound nuclear proteins were extracted by incubation with nuclease. To verify fractionation, 25 ml of each extract was analysed by Western blotting using specific antibodies against proteins from various cellular compartments, including for anti-HSP90 (cytoplasmic; Stratagene), calreticulin (membrane; Cell Signalling) and histone3 (chromatin-bound; Cell Signalling) antibodies.

#### Intracellular ROS, NADPH and superoxide assay.

To investigate intracellular ROS upregulation, we examined intracellular ROS levels after *ts*1 infection using the fluorescent probe 5- (and 6)-chloromethyl-2′,7′-dichlorodihydrofluorescein diacetate acetyl ester (CM-H_2_DCFDA) (Molecular Probe) to measure intracellular H_2_O_2_ levels. At the indicated times (4, 8, 24 and 48 h p.i.), *ts*1-infected astrocytes were incubated with CM-H_2_DCFDA for 30 min. DCFDA is deacetylated by endogenous esterases to DCF, which reacts with H_2_O_2_ to generate fluorescence. To determine NADPH levels in the cytoplasm, an NADPH Assay kit (BioVision) was used following the manufacturer’s instructions. Briefly, cells were trypsinized and washed with PBS following 100 mM apocynin treatment in the medium. NADPH was extracted from the cell pellets (5×10^5^ cells) and incubated with the enzyme mixture and colorimetric developer for 1 h at 37 °C. To detect O_2_^−^ levels in the CNS, DHE was injected into uninfected or 30 days p.i. *ts*1-infected mice, as described previously (Behrens *et al.*, 2007). Briefly, two serial intraperitoneal injections of freshly prepared DHE (27 mg kg^−1^; Molecular Probes) were given to the uninfected mice and to the 30 days p.i. *ts*1-infected mice, as described, at 30 min intervals. After 18 h, mice were perfused intracardially with cold saline followed by 4 % paraformaldehyde in PBS for immunohistochemistry experiments. The whole brain was incubated in 30 % sucrose solution overnight and frozen in optimal cutting temperature embedding medium (Sakura Finetek) for frozen sections.

#### Apocynin treatment of *ts*1-infected mice.

To test the effect of apocynin treatment on *ts*1-mediated neuropathogenesis, mouse pups at 2 days post-natal were infected with *ts*1 and divided into two groups. One group received normal saline and the other received freshly prepared apocynin (Sigma Aldrich) at 100 mg kg^−1^ (amount/body weight) delivered intraperitoneally for five continuous days per week, followed by two resting days. Mice from both groups were checked daily for clinical signs of disease and sacrificed when moribund and paralysed. All animal procedures were performed according to protocols approved by The University of Texas M. D. Anderson Cancer Center Institutional Animal Care and Use Committee.

#### Immunohistochemistry and immunocytochemistry.

For immunohistochemistry, frozen mouse brainstem tissues were sliced into 6 µm sagittal sections encompassing the brainstem region. Brainstem tissue sections were washed in PBS and incubated in 5 % normal donkey serum in PBS/0.1 % Triton X-100 for 1 h at room temperature. Primary antibodies [goat anti-GFAP: 1 : 200 (Santa Cruz Biotechnology), mouse anti-NeuN: 1 : 1000 (Covance) and rabbit anti-p30capsid: 1 : 500] were diluted in 5 % normal donkey serum in PBS and applied to the sections for 1 h at room temperature. The sections were then washed in PBS and incubated in a 1 : 500 dilution of Texas Red-conjugated donkey anti-goat IgG or FITC-conjugated donkey anti-mouse IgG antibody (Jackson ImmunoResearch) for 1 h at room temperature, and mounted sequentially in glass slides using ProLong anti-fade mounting reagent (Molecular Probes) containing DAPI (Molecular Probes).

For immunocytochemistry, uninfected control and *ts*1-infected C1 cells were placed on poly-l-lysine-coated coverslips and fixed in 4 % paraformaldehyde for 20 min and then washed with PBS. The fixed cells were incubated in PBS containing 0.05 % Tween 20 and 5 % donkey serum for 30 min and washed with PBS. The coverslips were then incubated with goat anti-gp70^env^ antibody and rabbit anti-SOD antibody followed by anti-goat IgG secondary antibody conjugated with Texas Red (Jackson ImmunoResearch) and anti-rabbit IgG secondary antibody conjugated with FITC (Jackson ImmunoResearch).

#### DNA and total RNA preparation and qPCR.

C1 astrocytes were infected with *ts*1 at an m.o.i. of 5. The infected cells were then divided into two groups: one group was not treated with any drug, and the other group was treated with 1 mM apocynin. mRNA was extracted using an RNeasy extraction kit and Oligotex, following the manufacturer’s instructions (Qiagen). Genomic DNA was purified using the genomic DNA extraction kit, following the manufacturer’s instruction (Promega) and treated with RNase to exclude RNA contamination. DNA was analysed for integrity using an Agilent 2100 Bioanalyser (Agilent Technologies). qPCR was subsequently performed using the ABI 7900HT Fast Real-time PCR system using SYBR Green master mix (Bio-Rad). DNA levels were normalized to the endogenous housekeeping gene glyceraldehyde-3-phosphate dehydrogenase (GAPDH). The primers specific to *ts*1 gPr80*^env^* were as described previously (Kuang *et al.*, 2010). Data analysis was performed using Sequence Detection System software from ABI, version 2.2.2. The experimental cycle threshold was calibrated against the GAPDH control product, and the ΔΔ*C*_t_ method was used to determine the amount of PCR product or genomic DNA purified from uninfected cells (0 %) and 24 h p.i. *ts*1-infected cells (100 %).

#### Statistics.

Data are presented as means±sem. Cell-culture experiments were conducted in triplicate or duplicate wells, with the means from three to four individual experiments used for statistical analysis. Statistical significance of the results was determined by Student’s *t*-test or low rank (Mantel–Cox) test and Gehan–Breslow–Wilcoxon test. Analyses of data were performed using Prism 5 Software (GraphPad Software).
